# Repaired coarctation of the aorta does not affect four-dimensional flow metrics in bicuspid aortic valve disease

**DOI:** 10.1093/icvts/ivae086

**Published:** 2024-05-04

**Authors:** Teemu Kiljander, Petteri Kauhanen, Saara Sillanmäki, Line Lottonen-Raikaslehto, Minna Husso, Elias Ylä-Herttuala, Petri Saari, Jorma Kokkonen, Jari Laukkanen, Pirjo Mustonen, Marja Hedman

**Affiliations:** Department of Cardiology, Tampere University Hospital, Heart Hospital NOVA, Jyväskylä, Finland; Institute of Clinical Medicine, University of Eastern Finland, Kuopio, Finland; Diagnostic Imaging Center, Kuopio University Hospital, Kuopio, Finland; Institute of Clinical Medicine, University of Eastern Finland, Kuopio, Finland; Diagnostic Imaging Center, Kuopio University Hospital, Kuopio, Finland; Diagnostic Imaging Center, Kuopio University Hospital, Kuopio, Finland; Diagnostic Imaging Center, Kuopio University Hospital, Kuopio, Finland; Diagnostic Imaging Center, Kuopio University Hospital, Kuopio, Finland; A.I. Virtanen Institute, University of Eastern Finland, Kuopio, Finland; Diagnostic Imaging Center, Kuopio University Hospital, Kuopio, Finland; Department of Cardiology, Tampere University Hospital, Heart Hospital NOVA, Jyväskylä, Finland; Institute of Clinical Medicine, University of Eastern Finland, Kuopio, Finland; Department of Medicine, Wellbeing Services County of Central Finland, Jyväskylä, Finland; Department of Cardiology, , Heart Center, Turku University Hospital, Turku, Finland; Institute of Clinical Medicine, University of Eastern Finland, Kuopio, Finland; Diagnostic Imaging Center, Kuopio University Hospital, Kuopio, Finland; Department of Cardiology, , Heart Center, Kuopio University Hospital, Kuopio, Finland

**Keywords:** Ascending aorta, Aortic coarctation, Bicuspid aortic valve, 4D flow MRI, Tricuspid aortic valve

## Abstract

**OBJECTIVES:**

The objective of this study was primarily to compare four-dimensional flow magnetic resonance imaging metrics in the ascending aorta (AA) of patients with right–left fusion type bicuspid aortic valve (RL-BAV) and repaired coarctation of the aorta (CoA) to RL-BAV without CoA. Metrics of patients with RL-BAV were also compared to the matched group of patients with common tricuspid aortic valve (TAV).

**METHODS:**

Eleven patients with RL-BAV and CoA, 11 patients with RL-BAV without CoA and 22 controls with TAV were investigated. Peak velocity (cm/s), peak flow (ml/s) and flow displacement (%) were analysed at 5 pre-defined AA levels. In addition, regional wall shear stress (WSS, mN/m^2^), circumferential WSS (WSS_c_) and axial WSS (WSS_a_) at all levels were quantified in 6 sectors of the aortic circle. Averaged WSS values on each level (WSS_avg_, WSS_c, avg_ and WSS_a, avg_) were calculated as well.

**RESULTS:**

Peak velocity at the proximal tubular AA was significantly lower in BAV and CoA group (*P* = 0.047) compared to BAV without CoA. In addition, the WSS_a, avg_ was found to be higher for the BAV and CoA group at proximal AA respectively (*P* = 0.040). No other significant differences were found between these groups. BAV group’s peak velocity was higher at every level (*P* < 0.001–0.004) compared to TAV group. Flow displacement was significantly higher for the BAV group at every level (*P* < 0.001) besides at the most distal level. All averaged WSS values were significantly higher in BAV patients in distal AA (*P* < 0.001–0.018).

**CONCLUSIONS:**

Repaired CoA does not relevantly alter four-dimensional flow metrics in the AA of patients with RL-BAV. However, RL-BAV majorly alters flow dynamics in the AA when compared to patients with TAV.

**Clinical trial registration number:**

https://www.clinicaltrials.gov/study/NCT05065996, Unique Protocol ID 5063566

## Graphical Abstract

**Figure:**
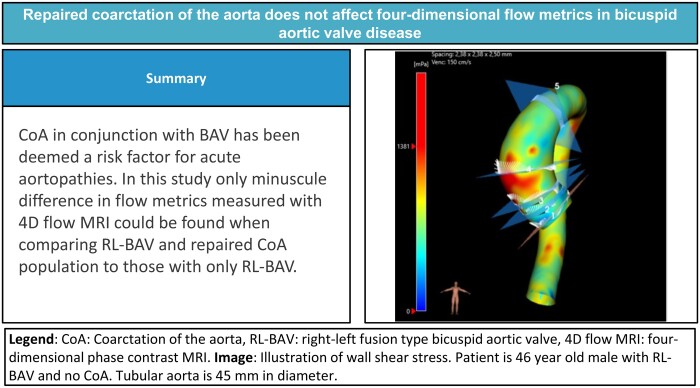

## INTRODUCTION

The bicuspid aortic valve (BAV) is the most common congenital cardiac malformation. The prevalence of BAV in the general population is 0.5–2% [1], and it is 3–4 times more common in males than in females [2]. BAV is a hereditary disease and has a 9% prevalence rate among first-degree relatives [3]. BAV is associated with higher risks of aortic valve regurgitation, stenosis and dilatation [4], which may lead to symptomatic cardiac diseases arising at an earlier age than in patients with tricuspid aortic valve (TAV) [5]. BAV morphologies are usually classified by which of the aortic valve leaflets have fused. The most common morphology is the fusion of the right and left coronary leaflets (RL, ca. 80%), followed by the right and noncoronary leaflets (RN ca. 17%), and, finally, the left and noncoronary leaflets (LN ca. 2%) [6, 7]. BAV is associated with coarctation of the aorta (CoA). About 7% of patients with BAV also have CoA, and, on the other hand, BAV can be found in ∼70–75% of patients with CoA [8]. The most serious complications of BAV-associated aortopathy are aortic dissection and the acute rupture of aortic aneurysm. However, such serious vascular events are commonly preceded by significant aortic dilatation, emphasizing the importance of screening BAV patients with aortopathies [9, 10].

To prevent the manifestation of acute, life-threatening aortopathies, BAV patients must undergo regular, lifelong follow-up visits at cardiology clinics. In the absence of any valvular disease, the follow-up visit frequency and surgery timing are solely based on the assessment of the aortic diameter without the knowledge of the effects of BAV on haemodynamic parameters [11]. Current imaging modalities of BAV and aortic diameters include transthoracic echocardiography, computed tomography and two-dimensional phase-contrast magnetic resonance imaging [11]. Novel tailored imaging modalities are required to assess the individual risk for aortic dilatation and associated acute aortopathies and subsequently allocate more frequent follow-ups to those with the highest risk. Repeated imaging follow-ups may not be needed for low-risk BAV patients. The recommended general cut-off threshold of ascending aorta (AA) diameter for prophylactic aortic surgery is 55 mm in BAV patients. A lower threshold of 50 mm is recommended for patients with additional risk factors, e.g. aortic coarctation [11].

One novel imaging method that fulfills the imaging requirements is time-resolved three-dimensional phase-contrast magnetic resonance imaging (4D flow MRI), which uses three-directional velocity encoding along the cardiac cycle. It thus allows for comprehensive evaluation and detailed visualization of the haemodynamic flow. Moreover, 4D flow MRI enables the study of advanced haemodynamic parameters such as wall shear stress (WSS), helicity, vorticity, flow angle, as well as assessment of turbulent and viscous energy loss. These help in characterizing complex flow patterns in the aorta. A particular advantage of 4D flow MRI is its ability to accurately position analysis planes at any location within the full volumetric coverage of the acquisition area [12, 13]. An increasing number of reports that compare 4D flow MRI against different clinically used imaging modalities have demonstrated altered flow parameters in populations with aortic dilatation and BAV [14–18]. Studies in patients with repaired CoA are, however, sparse. It is demonstrated that patients with repaired or mild CoA have a higher risk for ascending aortic complications despite their younger age and smaller aortic root diameter [19].

The primary aim of this study was to investigate whether haemodynamic metrics measured using 4D flow MRI in AA are affected by repaired CoA compared to patients with no CoA in association with right–left fusion type bicuspid aortic valve (RL-BAV). The 2nd aim was to compare the 4D flow MRI metrics between RL-BAV and TAV.

## MATERIALS AND METHODS

### Ethical statement

This study was approved by the Ethical Committee of the Northern Savo Hospital District (200/2017, 21 May 2019), and written informed consent was obtained from all participants. The study followed the Declaration of Helsinki, and all procedures were performed in accordance with relevant guidelines and regulations.

### Study population

The BAV patient registry originally consisted of 63 individuals recruited from the Central Finland Hospital District. To focus exclusively on RL fusion, we applied specific exclusion criteria: patients with LN or RN-fusion type (*n* = 15), those with valvular/aortic prostheses (*n* = 18), individuals with suboptimal imaging quality or missing imaging data (*n* = 6) and individuals with significant valvular stenosis (defined as a peak velocity exceeding 300 cm/s, as assessed by two-dimensional flow MRI) (*n* = 2) were excluded. Following these exclusions, our study formed a cohort comprising 11 RL-BAV patients who had undergone prior CoA repair and 11 RL-BAV patients with no history of CoA. Aortic MRI was performed at the Kuopio University Hospital, Kuopio, to include the possibility of 4D flow MRI. A control group of 22 patients with TAV and matching AA diameters was also included in the 4D flow MRI study.

### Clinical characteristics

Patients’ clinical characteristics, such as height, weight, sex and baseline diseases, were collected from the electronic medical records of the Central Finland Hospital District.

### Magnetic resonance imaging

A 1.5T Siemens MAGNETOM Aera (Siemens GmbG, Erlangen, Germany) was used to perform all the MRI scans. Contrast media was not used in any of the scans. The overall scan time, including the acquisition of anatomical and 4D flow data, was ∼45 min each. The MRI scan protocol has been described in more detail in a previous study by Kauhanen *et al.* [20].

### Four-dimensional flow magnetic resonance imaging

A standard Siemens ECG-gated 4D flow MRI sequence was acquired in free breathing and without contrast media. The 3D volume covering the entire AA was acquired and artefacts caused by respiratory motion were minimized by averaging. The imaging parameters of the 4D sequences were as follows: echo time 2.8 ms, repetition time 5.25 ms, number of segments 2, spatial resolution 2.3 × 2.3 × 3.0 ${\text{mm}}_{,}^{3}$ flip angle 7°, 18–25 cardiac phases and 15 slices. The VENC was set to the lowest non-aliasing velocity in the scout images.

### Assessment of the four-dimensional flow parameters

Flow quantification of the 4D flow MRI datasets were performed through CAAS MR 4D flow (Pie Medical Imaging, Maastricht, Netherlands) software using the CAAS MR 4D Artery module.

**Figure 1:**
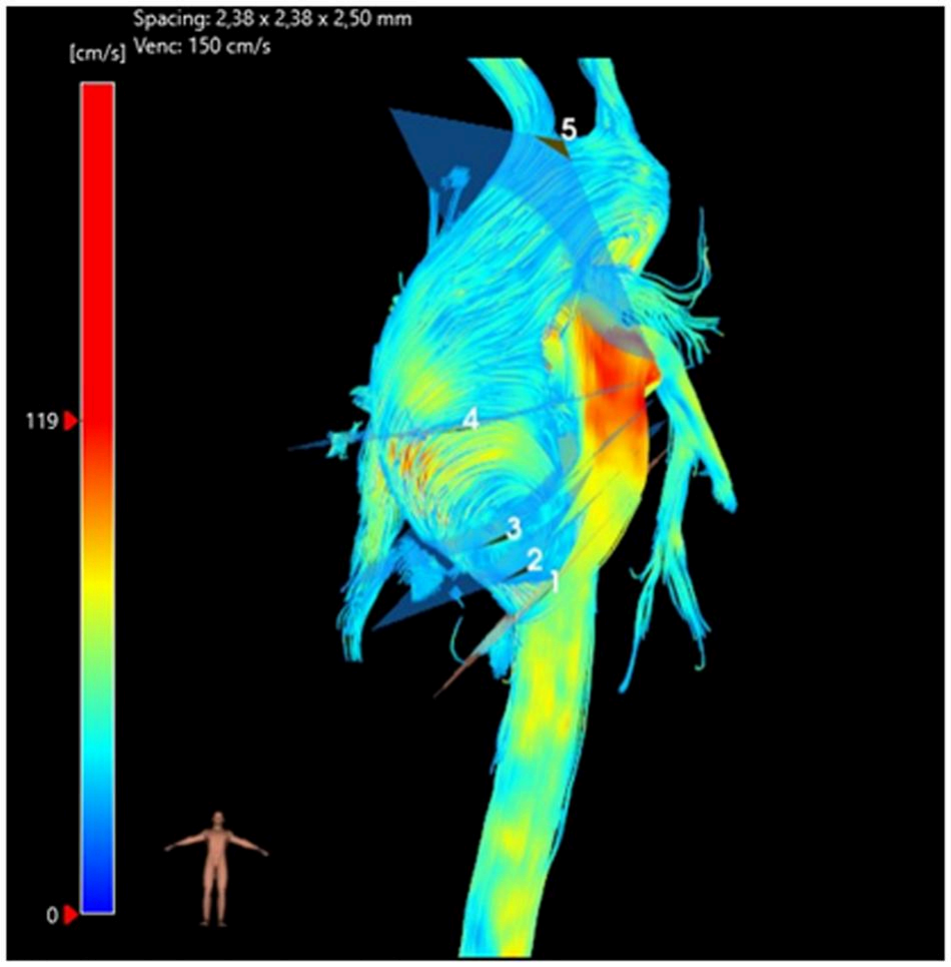
Illustration of 4D flow magnetic resonance imaging planes of measurement.

Flow parameters were evaluated at 5 predetermined planes in the AA as follows: sinus Valsalva, sinotubular junction, proximal AA, mid-AA and proximal aortic arch (Fig. 1). The following parameters were evaluated: peak velocity (cm/s), peak flow (ml/min), flow displacement (FD, %) and WSS (mN/m^2^).

In addition to WSS, circumferential WSS (WSS_c_, perpendicular to the centreline of the aorta) and axial WSS (WSS_a_, parallel to the centreline of the aorta) were also calculated. Circumferentially averaged WSS (WSS_avg_), WSS_c_ (WSS_c, avg_) and WSSa (WSS_a, avg_) were calculated on each plane, and the values in BAV subgroups were compared to each other and to the TAV group. The regional distribution of WSS across the aortic ring was further evaluated on planes 4 and 5, where the biggest difference in mean WSS between the BAV and TAV groups could be found.

**Figure 2:**
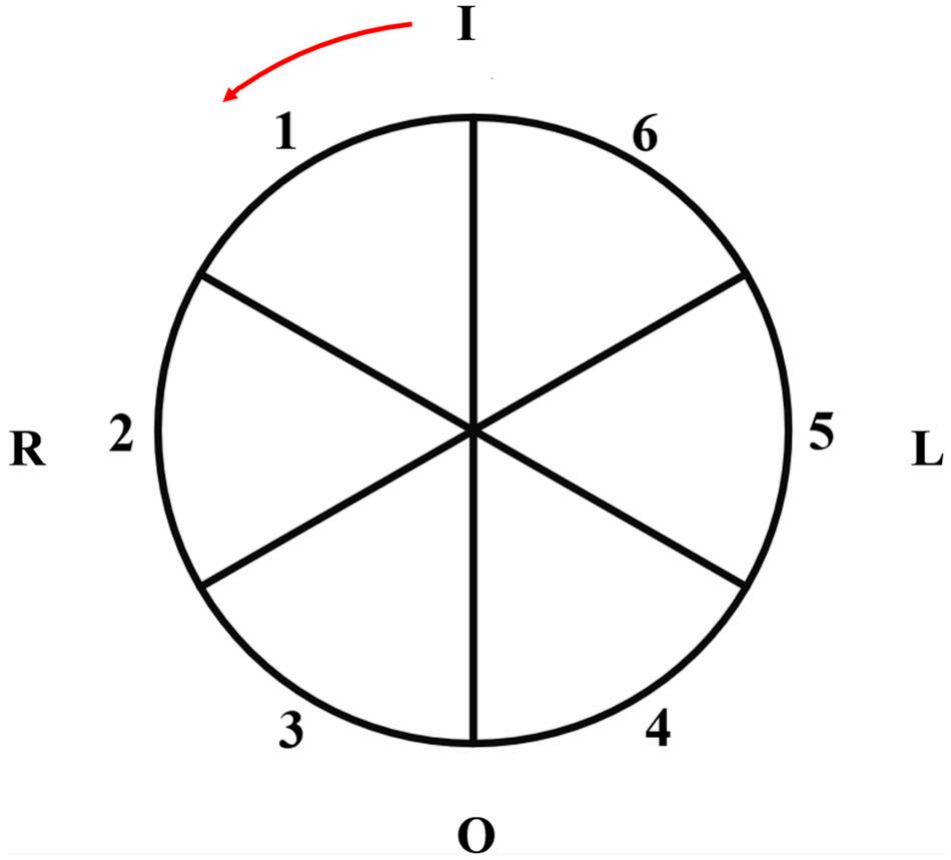
Aortic wall is divided into six 60-degree segments. I: inner curvature; L: left; O: outer curvature; R: right.

The WSS was calculated using the CAAS MR 4D flow software at 4° intervals on each plane. WSS_avg_ values were calculated by averaging the WSS on each plane. For further WSS regional analysis, the aortic wall was divided into 6 sectors, which accounted for 60° each. The starting point was set at the inner curvature of the aorta, and the sectors were numbered counterclockwise (1, 2, 3, etc.) (Fig. 2). The WSS was averaged in each sector.

FD was defined as the distance between the geometrical centre of the aorta and the centre of the forward flow divided by aortic diameter (%). The direction of the FD was visually assessed from two-dimensional in-plane images.

### Statistical analysis

Statistical analyses were performed using IBM SPSS Statistics (version 27). The baseline characteristics are presented as means ± standard deviations, which were calculated using the Student’s independent samples *t*-test. A *P*-value of <0.05 was considered statistically significant. The differences in baseline diseases (yes versus no) were calculated using the Chi-square test. Given the relatively small sample sizes and non-normally distributed data in our patient population, paired comparisons of flow metrics between BAV subgroups and BAV versus TAV groups were performed using the Mann–Whitney *U*-test. These results are presented as median (interquartile range).

## RESULTS

### Study population

A total of 44 patients were included in this study. Of these, 11 patients with RL-BAV and repaired CoA (BAV + CoA) and 11 patients with RL-BAV without CoA (BAV-CoA) were compared against each other. In further analyses, a pooled group of all 22 BAV patients was also compared with 22 TAV subjects.

The mean age in the BAV + CoA population (30.8 ± 10.1 years) was lower than in the BAV-CoA population (43 ± 13.2 years). There was no significant difference between the number of male and female subjects (Table 1). The maximum average diameter of the tubular aorta was also lower in the BAV + CoA group (32.6 ± 6.5 mm vs 39.3 ± 6.7 mm, *P *=* *0.028). There were no significant differences in the height, weight and body surface area of the populations, nor were there any differences in the prevalence of diabetes, hypertension, hypercholesterolaemia or coronary artery disease (Table 1).

**Table 1:** Characteristics of the study population

	BAV + CoA, *n* = 11	BAV–CoA, *n* = 11	*P*-value	BAV, *n* = 22	TAV, *n* = 22	*P-*value
	Mean ± SD	Mean ± SD		Mean ± SD	Mean ± SD	
Age (years)	30.8 ± 10.1	43.0 ± 13.2	**0.025**	36.9 ± 13.1	58.9 ± 6.2	**<0.001**
Height (cm)	171.4 ± 9.3	170.7 ± 9.2	0.87	171.1 ± 9.0	174.3 ± 6.3	0.18
Weight (kg)	72.0 ± 16.9	76.6 ± 13.2	0.48	74.32 ± 15.0	82.3 ± 13.3	0.068
BSA (m^2^)	1.84 ± 0.24	1.88 ± 0.19	0.62	1.85 ± 0.21	1.97 ± 0.16	0.057
Aortic root diameter (mm)	37.4 ± 6.2	40.9 ± 7.0	0.22	39.1 ± 3.2	38.1 ± 3.2	0.52
Tubular aorta diameter (mm)	32.6 ± 6.5	39.3 ± 6.7	**0.028**	35.9 ± 7.3	36.3 ± 5.3	0.83

	*n* (%)	*n* (%)		*n* (%)	*n* (%)	

Male	5 (45.5)	5 (45.5)	1.00	10 (45.5)	16 (72.7)	0.066
Diabetes	0	0	n/a	0	2	0.15
Hypertension	4 (36.4)	1 (9.1)	0.13	5 (22.7)	12 (54.5)	**0.030**
Hypercholesterolemia	1 (9.1)	1 (9.1)	1.00	2 (9.1)	6 (27.3)	0.12
Coronary artery disease	0	0	n/a	0	2 (9.1)	0.15

Bolded values indicate a statistically significant (*P* < 0.05) difference.

BAV: bicuspid aortic valve; BSA: body surface area; CoA: coarctation of aorta; *n*: the number of patients; SD: standard deviation, n/a: not applicable.

The mean age of the pooled BAV population (36.9 ± 13.1 years) was significantly lower than that of the TAV population (58.9 ± 6.2 years). Otherwise, the groups were matched in terms of sex, aortic diameter on sinus Valsalva and tubular levels, height, weight and body surface area. Hypertension was seen to be more prevalent in the TAV group (12 vs 5 cases, *P *=* *0.030). There was no significant difference in the prevalence of coronary artery disease, diabetes or hypercholesterolaemia. Detailed characteristics of the BAV versus TAV patient groups are presented in Table 1.

None of the patients with repaired CoA had significant re-coarctation, defined as >50% difference in diameter of the narrowest segment of the aorta, compared to the descending aorta at the diaphragm level.

### Peak velocity

Detailed peak velocity values in the different planes of AA are presented in Table 2. The peak velocity of the flow was lower in the BAV + CoA group on plane 4 [121.3 (101.8–147.3) cm/s] than in the BAV-CoA group [141.6 (131.9–179.6) cm/s, *P *=* *0.047]. However, no significant differences in peak velocity were observed in other planes of the aorta.

**Table 2:** Peak velocity (cm/s) in the different planes of ascending aorta presented as median (interquartile range)

	BAV + CoA	BAV–CoA	*P*-value	BAV	TAV	*P*-value
Plane 1	166.2 (153.7–196.6)	159.7 (123.6–189.7)	0.44	164.4 (133.4–190.1)	110.0 (103.7–123.1)	**<0.001**
Plane 2	168.2 (149.4–177.2)	156.0 (141.1–191.1)	0.97	165.6 (133.1–170.8)	139.0 (123.6–155.4)	**0.004**
Plane 3	149.2 (126.5–183.8)	158.9 (142.6–189.4)	0.41	154.0 (133.1–187.1)	122.1 (103.6–132.7)	**<0.001**
Plane 4	121.3 (101.8–147.3)	141.6 (131.9–179.6)	**0.047**	136.6 (110.6–170.8)	89.8 (83.1–99.5)	**<0.001**
Plane 5	104.2 (86.3–131.2)	115.3 (90.2–140.8)	0.56	105.7 (86.6–135.3)	74.2 (66.7–83.2)	**<0.001**

Bolded values indicate a statistically significant (*P* < 0.05) difference.

BAV: bicuspid aortic valve; CoA: coarctation of aorta; TAV: tricuspid aortic valve.

Peak velocities were significantly higher (*P < *0.001–0.004) in the BAV patients than in TAV patients on planes 1–5 (Table 2).

### Other flow parameters

No significant differences in peak flow in the AA were found among the BAV subgroups and between the BAV and TAV populations. There was no statistically significant difference in the FD amount between the BAV subgroups.

On the other hand, the FD was found to be higher in the BAV group compared to the TAV group in plane 1 [8.5 (4.0–11.0)% versus 3.0 (2.0–4.0)%, *P *<* *0.001], plane 2 [10.0 (7.0–12.3)% vs 2.0 (1.8–3.3)%, *P *<* *0.001], plane 3 [11.0 (10.0–14.0)% vs 2.0 (1.0–3.0)%, *P *<* *0.001] and plane 4 [12.5 (8.0–14.3)% vs 3.0 (2.0–4.0)%, *P *<* *0.001]. However, no differences in FD parameters were observed in ascending aortic plane 5.

**Figure 3:**
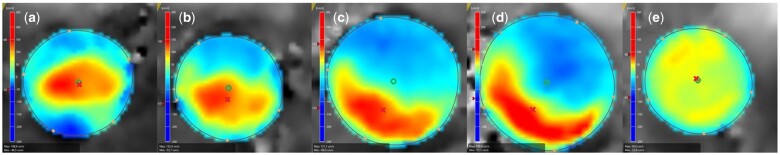
Illustration of flow displacement on planes 1–5. The circle indicates the geometrical centre of the aorta, and the cross indicates the centre of forward flow. (**a**) Valvular level, (**b**) sinotubular junction, (**c**) proximal AA, (**d**) mid-AA and (**e**) proximal arotic arch.

In most BAV-CoA cases, there was a tendency towards anterior displacement of the flow on plane 2, shifting more into the right-anterior side on planes 3–4, and back towards the anatomical centreline on plane 5. On the other hand, in most BAV + CoA cases, there was often slight anterior displacement of flow on plane 3, shifting to the right on plane 4. More heterogeneous directions of FD could be found in the BAV + CoA group (Fig. 3).

### Wall shear stress

#### Average wall shear stress

The only significant difference in WSS_avg_ between the different BAV subgroups was found in the WSS_a, avg_ on plane 3, where WSS_a, avg_ was detected to be higher in the BAV + CoA group [842 (583–1109) mN/m^2^] than in the BAV-CoA group [619 (480–714) mN/m^2^, *P* = 0.040].

In the BAV group, WSS_avg_ was found to be significantly lower in the aortic root (planes 1 and 2) than in the TAV group. WSS_a, avg_ on plane 1 and WSS_a, avg_ and WSS_c, avg_ on plane 2 were lower in the BAV group. A transition point in WSS_avg_ could be found on plane 3. WSS_c, avg_ was significantly higher in the BAV group while no difference in WSS_avg_ and WSS_a, avg_ could be found on this plane. Further up in the aorta, on planes 4 and 5, WSS_avg_, WSS_c, avg_ and WSS_a, avg_ were found to be significantly higher in the BAV group compared to the TAV group. Detailed WSS_avg_ values are presented in Table 3.

**Table 3:** Average wall shear stress (mN/m^2^) on planes 1–5 presented as median (interquartile range)

	BAV + CoA	BAV–CoA	*P*-value	BAV	TAV	*P*-value
**Plane 1**						
WSS_avg_	795 (583–1047)	591 (439–832)	0.30	654 (488–1047)	1002 (806–1277)	**0.004**
WSS_c, avg_	433 (264–708)	308 (206–252)	0.40	376 (209–555)	303 (237–456)	0.37
WSS_a, avg_	463 (373–892)	439 (320–583)	0.40	461 (370–699)	916 (742–1115)	**<0.001**
**Plane 2**						
WSS_avg_	1047 (940–1169)	1090 (862–1361)	0.70	1053 (921–1274)	1298 (1119–1451)	**0.012**
WSS_c, avg_	251 (230–331)	279 (179–413)	0.95	254 (181–352)	349 (261–426)	**0.037**
WSS_a, avg_	958 (890–1129)	978 (831–1218)	0.80	970 (847–1164)	1186 (1052–1365)	**0.011**
**Plane 3**						
WSS_avg_	1107 (916–1327)	944 (789–1058)	0.17	945 (816–1158)	956 (799–1094)	0.076
WSS_c, avg_	493 (355–666)	531 (407–611)	0.80	522 (391–662)	392 (329–458)	**0.006**
WSS_a, avg_	842 (583–1109)	619 (480–714)	**0.040**	688 (578–858)	828 (653–945)	0.18
**Plane 4**						
WSS_avg_	1010 (888–1261)	1086 (996–1312)	0.17	1072 (915–1274)	652 (533–737)	**<0.001**
WSS_c, avg_	584 (379–667)	601 (525–802)	0.30	591 (515–758)	239 (182–332)	**<0.001**
WSS_a, avg_	697 (631–922)	816 (726–977)	0.12	747 (686–955)	538 (428–656)	**<0.001**
**Plane 5**						
WSS_avg_	1031 (908–1216)	910 (769–1430)	0.80	942 (793–1274)	613 (554–701)	**<0.001**
WSS_c, avg_	592 (342–689)	510 (418–782)	0.95	519 (413–712)	168 (144–206)	**<0.001**
WSS_a, avg_	637 (583–999)	597 (565–1025)	0.75	628 (579–1005)	573 (503–695)	**0.018**

Bolded values indicate a statistically significant (*P* < 0.05) difference.

BAV: bicuspid aortic valve; CoA: coarctation of aorta; TAV: tricuspid aortic valve; WSS_a, avg_: average axial wall shear stress; WSS_avg_: average wall shear stress; WSS_c, avg_: average circumferential wall shear stress.

#### Regional wall shear stress

Further analysis of WSS between the BAV subgroups was undertaken to determine possible differences in the regional distribution of WSS across the aortic wall.

**Figure 4:**
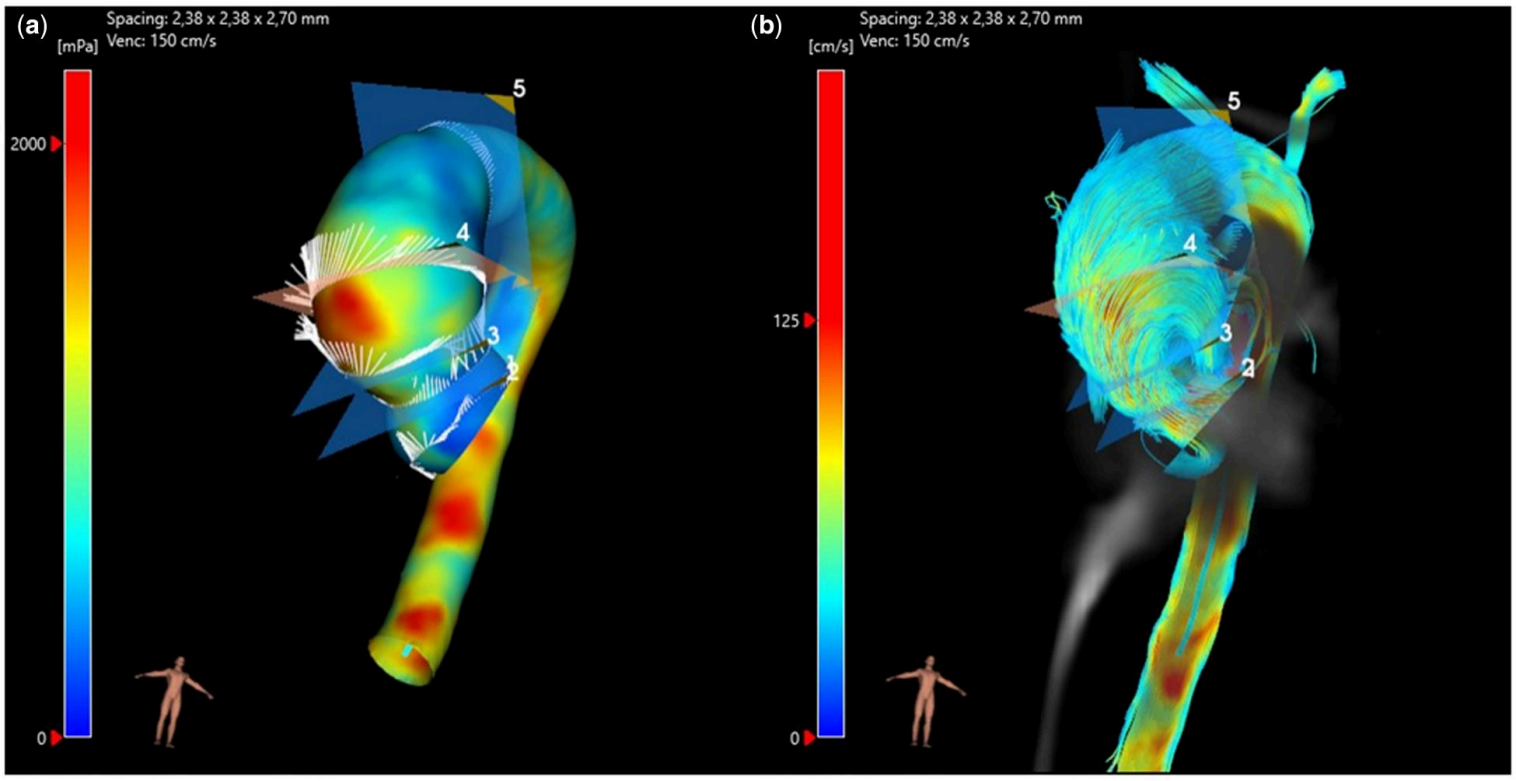
Illustration of (**a**) total WSS and (**b**) 4D flow. A 46-year-old female with BAV and without CoA.

When comparing WSS, WSS_c_ and WSS_a_ on a sector-by-sector basis (5 planes, 6 sectors on each plane) between the BAV subgroups, no significant differences could be found in any region’s WSS. On the contrary, when comparing the BAV and TAV groups, a significant difference was found in 56 of the 90 analysed WSS parameters. WSS distribution on plane 3 is provided in Table 4. An illustration of the total WSS and 4D flow is given in Fig. 4.

**Table 4:** Regional distribution of wall shear stress (mN/m^2^) on plane 3 presented as median (interquartile range)

	BAV + CoA	BAV–CoA	*P*-value	BAV	TAV	*P*-value
**Sector 1**						
WSS	1369 (806–1615)	1087 (773–1274)	0.22	1157 (798–1430)	1010 (811–1209)	0.12
WSS_c_	381 (254–479)	594 (433–731)	0.065	453 (310–690)	292 (188–467)	**0.017**
WSS_a_	1146 (739–1464)	589 (453–1183)	0.065	867 (513–1285)	910 (486–1166)	0.57
**Sector 2**						
WSS	1085 (1054–1481)	1122 (792–1291)	0.52	1118 (1000–1383)	1016 (975–1383)	0.36
WSS_c_	667 (390–860)	497 (299–686)	0.52	518 (341–686)	269 (167–380)	**0.002**
WSS_a_	786 (661–1154)	995 (554–1092)	0.80	869 (626–1108)	940 (791–1223)	0.26
**Sector 3**						
WSS	894 (782–1148)	946 (722–1020)	0.85	920 (747–1078)	830 (731–1107)	0.50
WSS_c_	508 (190–706)	505 (355–679)	0.65	506 (277–683)	387 (225–456)	**0.020**
WSS_a_	769 (430–929)	484 (369–857)	0.52	569 (394–875)	765 (656–1100)	0.082
**Sector 4**						
WSS	909 (528–1697)	723 (618–1033)	0.65	812 (552–1261)	1000 (764–1142)	0.22
WSS_c_	360 (207–682)	512 (298–684)	0.52	431 (277–683)	506 (280–719)	0.91
WSS_a_	710 (353–1330)	460 (357–637)	0.19	502 (356–948)	681 (514–987)	**0.013**
**Sector 5**						
WSS	1030 (602–1176)	771 (519–923)	0.19	878 (536–1121)	976 (862–1204)	0.12
WSS_c_	723 (258–918)	418 (191–761)	0.44	465 (248–847)	515 (382–654)	0.98
WSS_a_	656 (428–820)	486 (152–597)	0.15	571 (346–773)	822 (597–1136)	**0.009**
**Sector 6**						
WSS	1019 (520–1268)	801 (535–1121)	0.65	894 (531–1165)	856 (702–1031)	0.91
WSS_c_	435 (277–583)	502 (360–576)	1.00	468 (396–582)	308 (232–409)	**0.006**
WSS_a_	492 (422–1068)	342 (278–894)	0.24	444 (296–994)	751 (651–941)	0.074

Bolded values indicate a statistically significant (*P* < 0.05) difference.

BAV: bicuspid aortic valve; CoA: coarctation of aorta; TAV: tricuspid aortic valve; WSS: wall shear stress; WSS_a_: average wall shear stress; WSS_c_: circumferential wall shear stress.

## DISCUSSION

In this study, 4D flow metrics in the AA of patients with RL-BAV and CoA were compared to those of patients with RL-BAV without CoA using detailed MRI assessments of aortic flow parameters. Repaired coarctation appeared to preserve MRI-based 4D flow conditions in patients with BAV. Furthermore, the flow parameters of all BAV patients were compared with a control group of 22 TAV patients with matching AA diameters.

Despite numerous studies on aortic flow conditions in different types of BAV fusion being published, only a few addressed CoA [21, 22]. In this study, we compared 4D flow metrics in patients with RL-fusion type BAV and repaired CoA. This specific BAV fusion type was chosen because it covers over 80% of all fusion types, and the flow patterns of different fusion types vary.

The European Society of Cardiology guidelines [11] categorizes the CoA concomitant to BAV as a risk factor for aortic rupture. It advises a lower threshold for prophylactic surgery for these patients. In 2020, Duijnhouwer *et al.* [23] determined that repaired CoA was not associated with a higher risk of aortic syndromes in 499 BAV patients, 121 of whom had CoA. Our study further strengthens this finding. Corrected CoA does not alter 4D flow metrics in the AA, and repaired CoA thus may not be associated with increased risk of acute aortic syndromes. However, further research is needed to investigate the long-term aortic outcomes among patients with repaired CoA.

### Effect of repaired aortic coarctation on flow metrics

This study found no significant differences in most parameters between the BAV subgroups, which suggests that repaired CoA seems to be similar to unaffected aorta in terms of the intra-aortic flow conditions in the AA. We did not find any difference in peak flow in the AA of the BAV subgroups. However, the peak velocity was observed to be statistically significantly lower in the BAV + CoA group at the pulmonary artery level (plane 4). If the repaired CoA further upstream would affect peak velocity, the difference should have also been observed in plane 5 and the velocity ought to be higher than in the BAV–CoA group. It can be speculated that repair itself somehow optimizes the unfavourite effects of BAV in terms of flow. On the other hand, this sole difference in peak velocity could simply be due to the small sample size studied.

Unexpectedly, no difference in FD at any level between the BAV groups was found in our study. This indicates that repaired CoA effects do not proceed in a retrograde manner. Additionally, controversial findings were found in some other studies, where the most prominent FD in both BAV groups was at the level of the pulmonary trunk. In some previous studies, the FD in RL-BAV was most prominent in the proximal parts of the aorta and was seen to diminish further upwards [14, 18]. This can indicate that the flow is directed more diagonally in the proximal AA. On the other hand, it may also be due to different aortic flow metrics measurement methods.

WSS values between the BAV subgroups were for the most part identical across the whole AA. The WSS_a, avg_ was found to be statistically higher in the BAV + CoA group in the mid-tubular aorta (plane 3). This indicated that the reconstruction of the aortic arch might affect the preceding flow. However, when compared sector-by-sector, no differences in WSS, WSS_c_ or WSS_a_ could be found. In 2 previous 4D flow MRI studies, increased WSS_c_ was associated with the dilatation of AA and AA growth rate [20, 24]. A similar connection between WSS_a_ and the dilatation or growth of AA has not been established anywhere else.

### Effect of right–left fusion type bicuspid aortic valve on flow metrics

Despite several characteristic differences between the BAV and TAV groups, numerous significant observations, similar to previous studies [14–18], were observed regarding aortic flow. The higher peak velocities of blood flow in the BAV group at proximal and mid-AA were consistent with the findings of Rodríguez-Palomares *et al.* [18], who analysed 101 BAV (78 RL-BAV) patients and compared the results with those of TAV patients. Furthermore, in our study, the difference in peak velocity existed up to the distal part of AA to the brachiocephalic trunk level (plane 5). This was likely caused by higher peak velocities near the valvular level (164.3 ± 69.9 vs 128.5 ± 27.7 cm/s, BAV versus TAV, respectively) that reflected more distally into the aorta.

FD was found to be higher in the BAV group at planes 1–4, while no difference could be found at the brachiocephalic trunk level (plane 5). In their cohort of 15 RL-BAV patients, Mahedivia *et al.* [14] only found a significant difference at the sinotubular junction when compared to controls with similar aorta sizes. The study by Rodríguez-Palomares *et al.* [18] found significantly higher FDs in BAV patients at all sinotubular, mid-ascending and distal AAs. This study’s overall finding regarding higher FD in RL-BAV patients in AA was strictly in line with those of previous studies.

On the other hand, conflicting findings regarding WSS values in AA have been found in previous studies. Geeraert *et al.* [25] analysed 53 BAV patients (35 RL-BAV) and found the average WSS_a_ at the sinus valsalva level to be lower in the BAV group. No difference in mid or distal AA was found. The average WSS_c_ was found to be higher in all levels of tubular aorta. Furthermore, Rodríguez-Palomares *et al.* [18] found the average WSS_c_ to be significantly higher at every level of AA in the BAV group, while the WSS_a_ was lower at the same levels. However, no significant difference in total average WSS was found in their study. On the contrary, Mahadevia *et al.* [14] found the average WSS in their cohort of 15 RL-BAV patients to be higher for BAV patients at the sinotubular junction, mid-AA and distal AA when compared to TAV patients.

One of the differences between our current study and previous study results was that we found a lower total WSS_avg_ and lower or equal WSS_c, avg_ at the sinus Valsalva level compared to TAV patients. Further distally in the aorta, on planes 4 and 5, we found WSS_avg_ and WSS_c, avg_ to be higher in the BAV. This is in line with previous findings. In addition, WSS_a, avg_ was also higher in the BAV group. This is contrary to previous findings [14, 18, 25] where WSS_a_ was either lower or did not significantly differ from the TAV populations.

In our experience, the precise tracking of aortic borders when analysing the flow with CAAS 4D MRI software might occasionally be challenging in case of asymmetrical flow orifice and asymmetrical aortic root geometry related to BAV. This in part could explain the unintuitive finding of lower WSS values in the aortic root in the BAV group despite higher peak velocity of the flow.

Based on these conflicting results, it appears that AA flow conditions in BAV patients varies even in patients with similar types of fusion, and some other affecting factors can be behind this phenomenon. On the other hand, MRI flow measurement methods may be challenging and affect the flow assessment results. Further studies are required to establish the clinical significance of these varying findings.

### Limitations

The main limitation of this clinical study is that the patient sample size was relatively small, which affects the statistical significance and the reliability of the observed results. This limited sample size is primarily attributable to the rarity of CoA. Furthermore, the differences in the mean age of the patients in the different subgroups might impact the results. Furthermore, the data collection originates from a relatively small catchment area.

### Inter- and intraobserver reproducibility

Inter- and intraobserver reproducibility of the 4D flow parameters were thoroughly investigated and have been described in detail in a previous study [20]. Identical methods of AA image reconstruction and measurements were used in this study.

## CONCLUSION

The AA flow conditions are affected by the most common cusp fusion type of RL-BAV when compared to TAV. However, there were only modest differences that we have considered to be clinically non-relevant in the 4D flow metrics in AA of BAV patients with or without CoA. This finding suggests that repaired CoA might not be considered a permanent risk factor for acute aortic events. This study contributes to increasing the data on altered 4D flow metrics in the AA of BAV patients.

## DATA AVAILIBILITY

All relevant data are within the manuscript.

## ABBREVIATIONS

4D: Four-dimensional
AA: Ascending aorta
BAV: Bicuspid aortic valve
CoA: Coarctation of aorta
FD: Flow displacement
MRI: Magnetic resonance imaging
TAV: Tricuspid aortic valve
WSS: Wall shear stress
WSS_a_: Axial wall shear stress
WSS_c_: Circumferential wall shear stress
WSS_avg_: Circumferentially averaged wall shear stress
